# Network structure of family function and self-management in patients with early chronic kidney disease amid the COVID-19 pandemic

**DOI:** 10.3389/fpubh.2022.1073409

**Published:** 2023-01-10

**Authors:** Yi Cui, Tianqi Yang, Rong Li, Hua Wang, Shasha Jin, Na Liu, Xufeng Liu, Hongbao Liu, Yinling Zhang

**Affiliations:** ^1^Department of Nursing, Air Force Medical University, Xi'an, China; ^2^Department of Military Medical Psychology, Air Force Medical University, Xi'an, China; ^3^Department of Nephrology, The First Affiliated Hospital of Air Force Medical University, Xi'an, China; ^4^Department of Nephrology, The Second Affiliated Hospital of Air Force Medical University, Xi'an, China

**Keywords:** family function, self-management, network structure, chronic kidney disease, COVID-19

## Abstract

**Background:**

Family function plays a pivotal role in self-management among patients with early chronic kidney disease (CKD), which has been especially important during the COVID-19 pandemic. Previous studies have investigated the relationships between family function and self-management using total scores through self-report questionnaires while ignoring the different components in both family function and self-management. The specific objective of this study was to explore the network structure of family function and self-management at the component level.

**Methods:**

A total of 360 patients with early CKD from three tertiary hospitals were enrolled in our cross-sectional survey from September to December 2021 in China. Components of family function were measured by the Family Adaptation Partnership Growth and Resolve Index, and components of self-management were measured by the Chronic Kidney Disease Self-management Instrument. Network analysis was used to establish the network structure.

**Results:**

Edges across the community of family function and self-management were mainly positive. Edges between F3 “Growth” and M1 “Self-integration”, F2 “Partnership” and M3 “Seeking social support,” F5 “Resolve” and M3 “Seeking social support” were the strongest. F3 “Growth” had the greatest positive bridge expected influence of family function community (0.12), and M3 “Seeking social support” had the greatest positive bridge expected influence of self-management community (0.16).

**Conclusion:**

We explored the potential pathways between different components of family function and self-management among patients with early CKD during the COVID-19 pandemic and found fine-grained relationships between them. The two nodes F3 “Growth” and M3 “Seeking social support” may provide a new idea from the perspective of family function for interventions to improve self-management.

## 1. Introduction

Chronic kidney disease (CKD) is a lifelong and incurable disease with progressive damage to renal structure and function. With its characteristics of high incidence and mortality and low awareness and treatment rates, CKD has become a world public health problem that seriously threatens human health (1, 2). In recent years, the incidence of CKD has been increasing yearly, and the global incidence in the general population is 11–13% (3), while the incidence in the Chinese population has increased from 10.8% in 2012 to 13.4% in 2017 (4). During the outbreak of coronavirus disease 2019 (COVID-19), patients with CKD have an increased risk of infection, resulting in acute kidney injury and various complications (5). Once an individual with CKD misses the early intervention period, he or she can only rely on dialysis or kidney transplantation to maintain survival after entering end-stage disease (6). Until that time, treatment is more expensive, quality of life is greatly reduced, and life span is cut short (7). Therefore, intervention in the early stage of CKD and delaying entry into end-stage disease are the top priorities in the management of CKD. In addition, during the pandemic, early intervention mainly depended on patients' self-management. Therefore, improving patients' self-management abilities is an important means to delay the progression of disease, which should be a major problem to study (8).

Self-management behavior refers to early CKD patients actively participating in their own health care activities, managing their emotions, diet, exercise and medication scientifically and effectively to improve the health outcomes of chronic diseases (9). It is a complex concept composed of self-integration, problem solving, seeking social support, and adherence to recommended regimen (10). At present, most studies mainly focus on the close relationship between the self-management of patients with CKD and their own self-perceived burden and self-efficacy (11, 12). There are also a small number of studies about patients who have diabetes and hypertension that found the better the family function is, the higher the patient's self-management ability (13, 14). During the COVID-19 pandemic, hospitals have strengthened prevention and control, requiring nucleic acid reports within 48 h. Cities have restricted travel due to high-risk areas, resulting in a decline in the number of visits. Only some critically ill patients need to go to the hospital and patients with early CKD can consult through internet hospitals (15). Most patients spent more time treating and managing their disease at home, which means that more scientific self-management is needed to effectively control the development and maintain the stability of the disease (16, 17). Therefore, how to improve patients' self-management ability from the perspective of family function has become at focus of this study during the current pandemic.

Family function means that family members provide financial, mental, and health care for each other so that patients with early CKD can get the care and understanding they need from the material, information, and emotional support provided by their families (18). Family function is a multidimensional concept, including the effectiveness of emotional connection, communication, family relationship regulation and joint response to external events (19). According to Bandura's cognitive theory, psychosocial factors, including family function, may play an important role in self-management (20). Especially when a pandemic breaks out, people must be isolated at home and get along closely with their families 24 h a day; thus, the relationship with their families changes dramatically from the usual state. At this time, the influence of family function on patients' self-management is even more prominent. Some studies have found that when patients' individual behavioral strength is combined with a cohesive functional family unit, patients' self-management is more active (21). A high degree of family care can also motivate patients to maintain long-term and effective health behaviors and continuously improve self-management (22). However, all the above studies regard self-management and family function as a whole and analyze them through their total scores, ignoring that both have complex structures. Ignoring the different roles played by different components of family function may obscure the most effective and scientific interventions to improve self-management. Therefore, to further clarify how family function is related to the various components of self-management, a more fine-grained approach is needed.

Network analysis is an innovative statistical method for complex variables that plays an important role in exploring the finer-grained correlation paths of two related variables. It is data-driven and can digitally analyze and visualize the relationship between various complex variables (23, 24). The network structure includes nodes and edges. Nodes represent observed variables, and edges represent the statistical relationship between observed variables, that is, the partial correlation coefficient between the two nodes after controlling for all other variables (25, 26). Compared with simple correlation statistical methods, the network analysis method can identify the more central or more important variables in the network (27). In addition, network analysis can also help us find the most interrelated variables (such as bridging variables) (28). When these variables are activated, it is more likely that the impact will be propagated throughout the network by connecting more edges, thus making the intervention more targeted. Therefore, it is feasible and scientific to apply network analysis to this study. First, the existing research is mainly based on the latent variable model that examines the relationship between family function and self-management at the total level while ignoring the deeper relationship between the two variables at the component level (29, 30). Both family function and self-management are complex systems composed of different dimensions. Treating each of their dimensions as a node can identify the specific relationship between the two components and enrich the existing studies. Second, spurious correlations between variables easily appear when there are many variables in psychological research. However, network analysis uses a partial correlation network to fit the data and uses a regularization process to control the occurrence of such false correlations, which can reduce the occurrence of related false positive correlations (31). Finally, network analysis can find the variables bridging the two communities of family function and self-management in the network through the bridge expected influence index (32). It provides an important intervention target for improving patients' self-management. In general, network analysis enables this study to explore the new close relationship between family function and self-management at the component level among patients with early CKD from a new perspective.

Different from previous studies that only focus on the single level of family function and self-management, this study relies on the advantages of network analysis to put all dimensions of family function and self-management into the same network. Based on previous studies that family function was the influencing factor of self-management, we hypothesized that the edges between family function and self-management are primarily positive and there exists important nodes in the network to improve self-management among patients with early CKD during the COVID-19 pandemic. This study has two objectives. First, we wanted to examine the potential pathways between different components of family function and self-management. Second, we use the bridge expected influence to test the strongest relationship between self-management and family function from the component level. Through the study of network analysis, we tried to deepen the understanding of the complex relationship between family function and self-management during the COVID pandemic, and provide scientific and theoretical support for intervention measures to improve family function and self-management.

## 2. Materials and methods

### 2.1. Ethics statement

The Ethics Committee of the Second Affiliated Hospital of Air Force Medical University approved this study (No. 202206-02). We explained the purpose and the informed consent to the participants, and after they agreed to participate, they finished the structural questionnaire anonymously in the ward by themselves. They could withdraw from the investigation at any time for any reason.

### 2.2. Participants and procedures

Three hundred sixty patients with early CKD from the departments of Nephrology in three tertiary hospitals in China were recruited for this cross-sectional survey through a convenience sampling technique from September to December 2021. The inclusion criteria were patients who (1) were aged ≥18 years; (2) met the diagnostic criteria of chronic kidney disease in the clinical practice guidelines of the Kidney Disease Outcomes Quality Initiative of the National Kidney Foundation; (3) were in clinical stages 1–3; and (4) had clear consciousness and normal communication skills and were able to complete the questionnaire. The exclusion criteria were patients who (1) had cognitive dysfunction or mental illness; (2) had complications with serious cardiovascular, nervous system, lung and other systemic diseases; and (3) had poor compliance. All participants were informed and voluntarily participated in this study. The sample size of participants calculated based on Kendall's (33) sample estimation should be 5–10 times that of the independent variable. There were 24 variables in this study, so the sample size was estimated to be 120–240. Assuming 20% of questionnaires would be invalid, the final sample size needed to be at least 144.

The researchers in charge of this study gave unified training to two nurses in the department of nephrology with the consent of the head nurse and they were responsible for data collection. . In the ward, researchers and trained nurses used unified instructions to introduce themselves to the patients who met the inclusion and exclusion criteria and explain the purpose and significance of this survey, as well as the matters needing attention and confidentiality principles of filling in the questionnaire. Patients completed the questionnaires anonymously. After the questionnaire was completed, the researchers took it back on the spot and checked its validity. Finally, a total of 391 patients participated in the study, and 360 valid questionnaires were collected for an effective rate of 92.07%.

### 2.3. Measures

#### 2.3.1. Basic information

The sociodemographic and disease data questionnaire was self-developed and included age, nation, gender, marital status, education level, occupation, residence, medical costs, monthly income (RMB), disease duration, review time, BMI index and CKD stages.

#### 2.3.2. Components of family function

The Family Adaptation Partnership Growth and Resolve (APGAR-family) Index is a measurement tool used to quickly detect a family member's evaluation of the family function, which reflects the subjective satisfaction degree of family members with the family function. It was developed by Smilkstein (34) and is composed of five items (adaptation, partnership, growth, affection and resolve) with a 3-point scale ranging from 0 (hardly never) to 2 (almost always). The Chinese version was introduced and translated in 1995, and it is widely used in various patients of all ages with good reliability and validity (35). The total score ranges from 0 to 10, with a higher score reflecting better family function. A total APGAR score of 0–3 suggests severe family dysfunction, 4–6 indicates moderate family dysfunction, and 7–10 indicates good family function. With its fast and effective characteristics, the questionnaire is widely used in clinical screening, treatment and research, which can help doctors determine the possible aspects of patients' family problems and serve as one of the indicators for treatment. The Cronbach's α of APGAR in this study was 0.929.

#### 2.3.3. Components of self-management

The Chronic Kidney Disease Self-management Instrument, which has been recognized internationally and widely used to measure the self-management behavior of patients with early CKD in stages 1–3, was developed by Lin et al. (36). The Chinese version was revised by Liu et al. (37), and it includes the four dimensions of self-integration, problem solving, seeking social support and adherence to the recommended regimen. The scale contains 29 items, and each item is measured on a 4-point Likert scale. The higher the score is, the stronger the patients' self-management ability. The score rate of the scale = actual score/highest possible score^*^100%, the score rate < 60% is low level, 60–80% is medium level, and ≥ 80% is high level. The Cronbach's α of the scale in this study was 0.956.

### 2.4. Data analysis

#### 2.4.1. Descriptive statistics

The SPSS 26.0 program (SPSS Inc., Chicago, IL) was used to analyze the data. Descriptive statistics are presented as the mean, standard deviation (SD), frequency, and percentage (%). Independent-sample *t*-test and one-way analysis of variance (ANOVA) were used to perform the comparison of family function and self-management among different sociodemographic subgroups. The significance test level was *P* < 0.05.

#### 2.4.2. Construction and evaluation of the network model

The R packages qgraph (38) and bootnet (39) were used to construct and evaluate the network. Statistical control in the network eliminated the interference of other nodes on the partial correlation of each node pair (31). By shrinking all edges and making inessential edges zero weight, the combination of the least absolute shrinkage and selection operator (LASSO) (40) regularization and Extended Bayesian Information Criterion (EBIC) (41) helped build a stable and understandable network. The hyperparameter of the EBIC was set to 0.5, and the Spearman correlation method was used. The nodes in the network are grouped into two communities, namely, the family function community (adaptation, partnership, growth, affection, and resolve) and the self-management community (self-integration, problem solving, seeking social support and adherence to the recommended regimen). In the evaluation of the network, the accuracy of edge weights was estimated by using non-parametric bootstrapping (1,000 bootstrapped samples), and a narrow 95% confidence interval of edge weights suggests good accuracy; the significance test of the difference in edge weight indices of different node pairs was conducted by using bootstrapping (1,000 bootstrapped samples, α = 0.05).

#### 2.4.3. Calculation and evaluation of bridge expected influence

The R packages networktools (32) and bootnet (39) were used to calculate and evaluate the bridge centrality of nodes. The bridge expected influence (BEI) of a node is defined as the sum of edge weights between the node and all nodes in other communities. BEI is especially suitable for networks with positive and negative edges, and a higher BEI means greater influence on other communities. In the evaluation of BEI, stability was tested by using case-dropping bootstrapping (1,000 bootstrapped samples) and quantified by the correlation stability coefficient (CS-coefficient). The CS-coefficient of acceptable stability was larger than 0.25. The significance test of the difference in BEI indices of different nodes was conducted by using bootstrapping (1,000 bootstrapped samples, α = 0.05).

## 3. Results

### 3.1. Descriptive statistics and comparison of family function and self-management

The mean age of 360 patients with early CKD (61.11% male) was 46.33 ± 16.54 years (mean ± SD, range 18–90 years). For family function, there were statistically significant differences in age (*F* = 3.443, *P* = 0.033), education level (*t* = −3.128, *P* = 0.002), and disease duration (*t* = −2.924, *P* = 0.004). For self-management, there were statistically significant differences in age (*F* = 17.781, *P* < 0.001), marital status (*F* = 2.741, *P* = 0.043), education level (*t* = −5.092, *P* < 0.001), occupation (*F* = 5.322, *P* = 0.005), residence (*t* = 2.954, *P* = 0.003), medical costs (*t* = −2.092, *P* = 0.041), monthly income (*F* = 3.717, *P* = 0.025), disease duration (*t* = −3.292, *P* < 0.001), and review time (*F* = 8.018, *P* < 0.001). Table 1 shows the sociodemographic descriptive statistics and comparison of family function and self-management.

**Table 1:** Sociodemographic descriptive statistics and comparison of family function and self-management (*N* = 360).

**Variable**	***n* (%)**		**Family function (M ±SD)**	**Self-management (M ±SD)**
**Age (years)**				
18~45	182 (50.56%)		7.31 ± 2.75	92.38 ± 17.26
46~69	144 (40.00%)		6.44 ± 3.24	80.65 ± 20.87
70~	34 (9.44%)		6.79 ± 3.31	78.85 ± 22.33
		*F*	3.443	17.781
		*P*	0.033	< 0.001
**Marital status**				
Unmarried	63 (17.50%)		6.71 ± 2.65	91.95 ± 15.33
Married	278 (77.22%)		6.97 ± 3.09	85.01 ± 21.25
Divorced	8 (2.22%)		6.38 ± 3.89	95.75 ± 11.74
Widowed	11 (3.06%)		7.00 ± 3.10	83.18 ± 13.81
		*F*	0.211	2.741
		*P*	0.889	0.043
**Education level**				
Junior college and below	292 (81.11%)		6.73 ± 3.17	84.24 ± 20.48
College	68 (18.89%)		7.72 ± 2.13	95.71 ± 15.72
		*t*	−3.128	−5.092
		*P*	0.002	0.000
**Occupation**				
On job	267 (74.17%)		6.91 ± 2.95	88.18 ± 19.17
Unemployment	36 (10.00%)		7.39 ± 2.72	85.44 ± 19.07
Retired	57 (15.83%)		6.63 ± 3.52	78.74 ± 23.60
		*F*	0.691	5.322
		*P*	0.502	0.005
**Residence**				
Urban	308 (85.56%)		6.88 ± 3.04	85.71 ± 21.03
Rural	52 (14.44%)		7.12 ± 2.94	90.54 ± 13.21
		*t*	1.232	2.954
		*P*	0.219	0.003
**Medical costs**				
Medical insurance	324 (90.00%)		6.94 ± 3.06	85.83 ± 20.57
Self-paying	36 (10.00%)		6.69 ± 2.70	91.64 ± 15.19
		*t*	0.458	−2.092
		*P*	0.647	0.041
**Monthly income (RMB)**				
< 2,000	99 (27.50%)		6.59 ± 3.22	84.80 ± 19.26
2,000~5,000	189 (52.5%)		6.87 ± 2.98	85.06 ± 20.74
>5000	72 (20.00%)		7.49 ± 2.82	92.15 ± 18.99
		*F*	1.901	3.717
		*P*	0.151	0.025
**Disease duration**				
3~12 months	266 (73.89%)		6.67 ± 3.17	84.36 ± 20.62
>12 months	94 (26.11%)		7.61 ± 2.47	92.21 ± 17.61
		*t*	−2.924	−3.292
		*P*	0.004	< 0.001
**Review time**				
< 1 month	118 (32.78%)		7.40 ± 2.80	90.81 ± 17.09
1~3 months	116 (32.22%)		6.71 ± 2.93	87.84 ± 18.70
>3 months	126 (35.00%)		6.65 ± 3.27	80.96 ± 22.84
		*F*	2.276	8.018
		*P*	0.104	< 0.001

A total of 42.22% of the patients with early CKD had moderate or severe family dysfunction, and 54.17% of patients' self-management level was moderate or low. Table 2 shows the mean scores, standard deviations and BEIs for each item in the network.

**Table 2:** Mean scores, standard deviations and BEIs for each item in the network (*N* = 360).

**Item**	**M**	**SD**	**BEI**
Family function	6.91	3.03	
F1. Adaptation	1.34	0.70	0.01
F2. Partnership	1.34	0.70	0.05
F3. Growth	1.40	0.68	0.12
F4. Affection	1.36	0.69	0.09
F5. Resolve	1.48	0.66	0.08
Self-management	86.41	20.16	
M1. Self-integration	34.44	8.33	0.13
M2. Problem solving	26.38	7.89	0.00
M3. Seeking social support	13.24	4.44	0.16
M4. Adherence to recommended regimen	12.34	3.20	0.06

### 3.2. Network structure of family function and self-management

**Figure 1:**
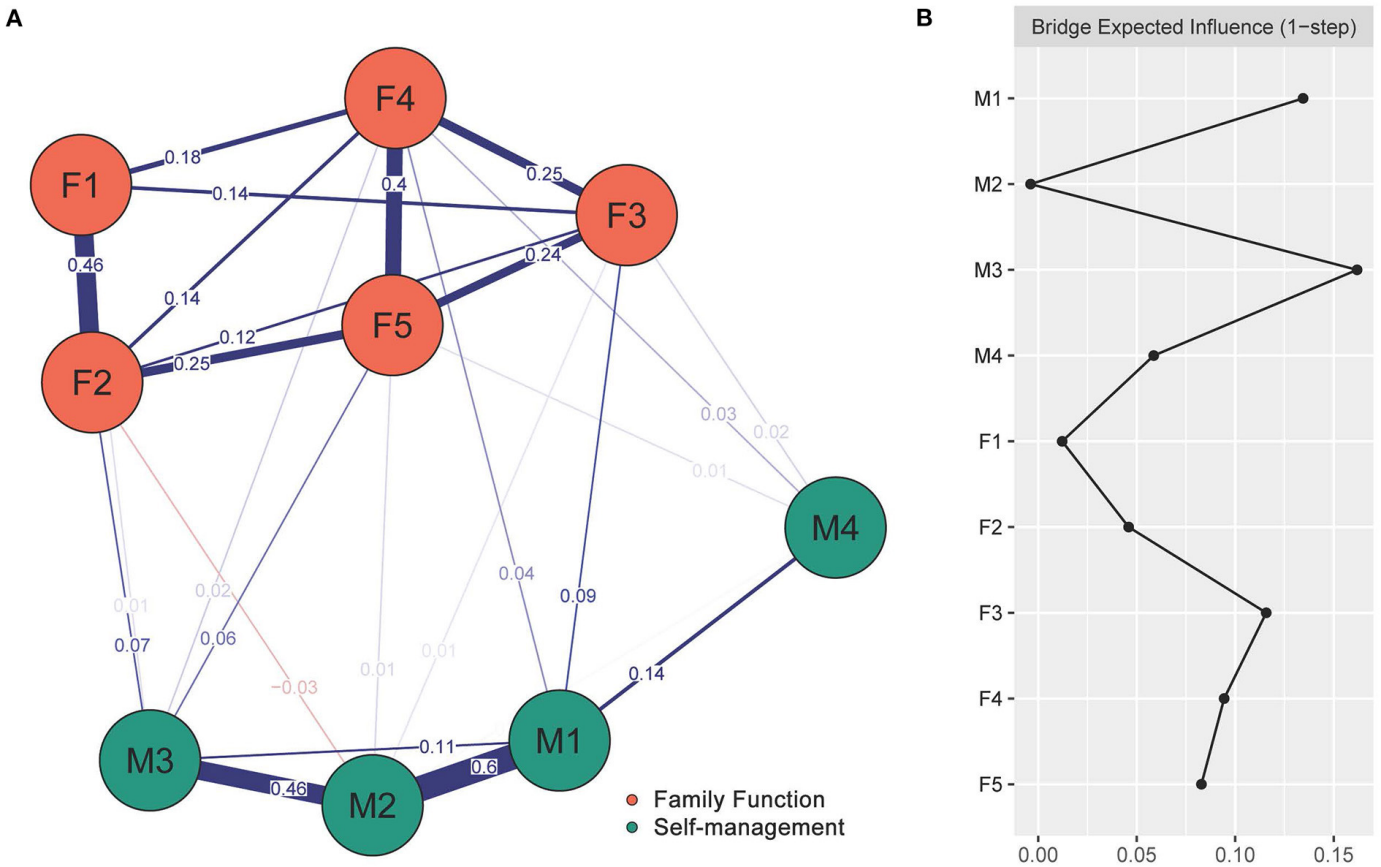
The network model of family function and self-management and the bridge expected influence. **(A)** The network model of family function and self-management. The red edges indicate negative partial correlations, the blue edges indicate positive partial correlations, and thick lines and saturated colors indicate large partial correlation coefficients. **(B)** The bridge expected influence indices in the network of family function and self-management (raw score). F1, Adaptation; F2, Partnership; F3, Growth; F4, Affection; F5, Resolve; M1, Self-integration; M2, Problem-solving; M3, Seeking social support; M4, Adherence to recommended regimen.

The network model of family function and self-management is presented in Figure 1A. Twelve edges across the communities of family function and self-management are non-zero (ranging from −0.03 to 0.09). In the cross-community edges, F1 “Adaptation” is positively correlated with M3 “Seeking social support” (edge weight = 0.01); F2 “Partnership” is negatively correlated with M2 “Problem solving” (edge weight = −0.03) and positively correlated with M3 “Seeking social support” (edge weight = 0.07); F3 “Growth” is positively correlated with three nodes of the self-management community, namely, M1 “Self-integration,” M2 “Problem solving” and M4 “Adherence to recommended regimen,” the strongest correlation was with M1 “Self-integration” (edge weight = 0.09); F4 “Affection” is positively correlated with three nodes of the self-management community, namely, M1 “Self-integration” M3 “Seeking social support” and M4 “Adherence to recommended regimen,” the strongest correlation was with M1 “Self-integration” (edge weight = 0.04); F5 “Resolve” is positively correlated with three nodes of the self-management community, namely, M2 “Problem solving” M3 “Seeking social support” and M4 “Adherence to recommended regimen,” the strongest correlation was with M3 “Seeking social support” (edge weight = 0.06). The correlation matrix of the network of family function and self-management can be found in Supplementary Table 1.

There was a narrow 95% confidence interval of edge weights in the network of family function and self-management, indicating good accuracy in the evaluation of edge weights (see Supplementary Figure 1). The result of the significance test of the difference in edge weight suggested that, generally, the edge weight within a community was larger than that across communities (*P* < 0.05, see Supplementary Figure 2).

### 3.3. Bridge expected influence

**Figure 2:**
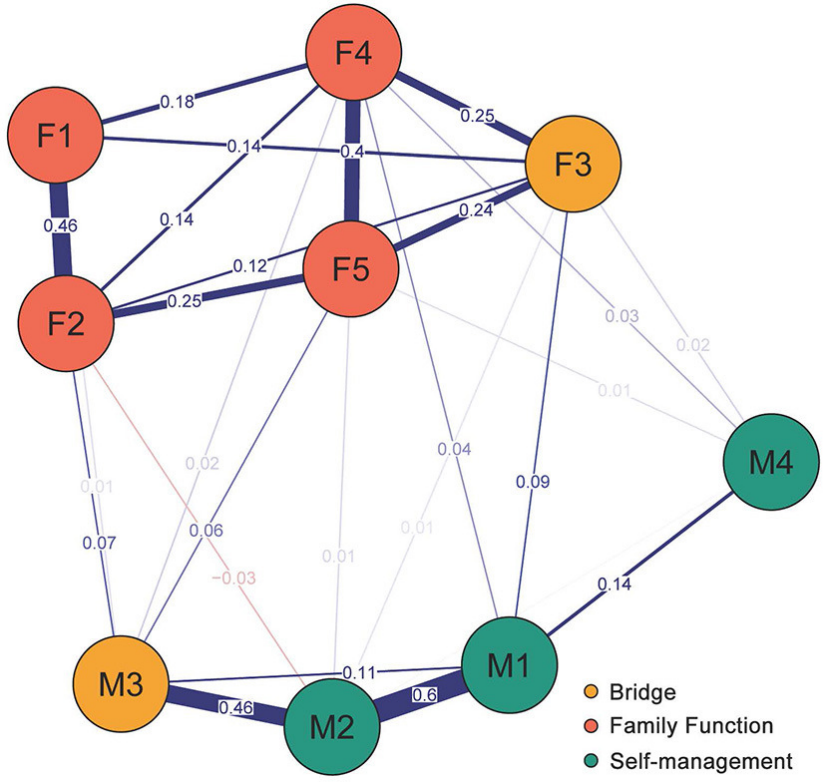
Network structure of family function and self-management showing bridge components in patients with early CKD.

As shown in Figure 1B, the results of BEI indices in the network of family function and self-management suggested that F3 “Growth” had the greatest positive bridge expected influence of family function community (0.12) and M3 “Seeking social support” had the greatest positive bridge expected influence of self-management community (0.16). These two nodes are regarded as the bridges connecting the two communities and are highly marked in the network, as shown in Figure 2. As the result of the BEI stability test suggested, the average correlation coefficient between the BEI indices of the subsample and the original sample declined gently with the reduction of the sampling proportion (see Supplementary Figure 3), and the CS-coefficient of the BEI was 0.36, which indicated acceptable stability. As the result of the significance test of the difference in BEI indices suggested, the BEI of M3 “Seeking social support” was statistically larger than that of M2 “Problem solving,” and the BEI of F3 “Growth” was statistically larger than that of F1 “Adaptation” (*P* < 0.05, see Supplementary Figure 4).

## 4. Discussion

Taking advantage of network analysis, we explored the potential pathways by which different components of family function are related to components of self-management among patients with early CKD. The results show that different components of family function may play a unique role in maintaining and improving patients' self-management. In a sense, our findings enrich the theoretical support for family-level interventions to improve self-management.

Within the family function and self-management network, most across-community edges were positive, and only one edge was negative. This is consistent with the results of previous studies on family function and self-management of patients with chronic disease. Patients with good family function have higher self-management ability (42). F1 “Adaptation” in the community of family function had a positive correlation with M3 “Seeking social support” in the community of self-management. This finding fully confirms previous studies showing that good adaptation can help patients seek social support as a coping strategy (43). Adaptation refers to what resources family members can obtain from the family when they encounter difficulties or crises and whether they can help them solve problems (35). CKD cannot be cured, and the progression of the disease can only be delayed through clinical treatment and self-management. Patients struggle with the disease for a long time. In this process, if a family can provide patients with sufficient resources and help them cope with the disease, patients will be more willing to seek social support on their own (44). This suggested that when carrying out health education for patients' families, medical staff should educate them to help patients as much as possible when patients encounter adversity, provide patients with physical and psychological resources, and lay a good foundation for patients to seek social support. F2 “Partnership” had a positive correlation with M3 “Seeking social support” and a negative correlation with M2 “Problem solving,” which is consistent with previous studies to some extent (45). Partnership refers to the way in which family members share responsibilities, solve problems and make decisions (35). When the family members of patients with early CKD cooperate with them to manage the disease, for example, the treatment plans sometimes need to be changed according to the progress of the disease, and the family is willing to take the initiative to help patients make treatment plans and medical decisions; the cost of treatment is also a burden for patients, but if family members share it with them, it can help relieve some of the pressure on patients (46). These are daily behaviors with a high degree of partnership with family members, which encourage patients to use social support to improve self-management (47). However, some scholars believe that some patients always rely too much on others to share responsibility without taking initiative to face their problems and always think about getting something for nothing or passing the buck (48). In contrast, this will cause negative effects, resulting in problems that cannot be dealt with well. It also suggests that moderate and correct partnership among families can help patients improve their self-management to the maximum extent through social support. Medical staff should educate family members to actively participate in the patients' self-management and share the difficulties encountered by them. Edge F3 “Growth” and M1 “Self-integration” revealed the strongest link. Self-integration is developed through interactive support with family members, and personal growth includes the improvement of self-integration, which is consistent with Divna Haslam's findings (49). Growth refers to the patients receiving family support and guidance on physical and mental development (35). In the long-term struggle against CKD, patients need to get along with their families for a long time, which can better reflect the role of family members' full support, correct information guidance and supervision, which can reduce family conflicts and improve patients' enthusiasm for self-growth and self-integration (50). This also suggests that, especially due to the pandemic, patients are highly volatile and impulsive and are prone to all kinds of emotional problems (51, 52). At this time, it is particularly important for family members to guide patients to learn self-integration, such as developing personal hobbies, learning to vent and make reasonable plans together. F4 “Affection” had the strongest positive correlation with M1 “Self –integration.” This is consistent with the study that the higher the degree of family participation is, the more care for the patient, and the better the patients' regulation strategy (53). Affection refers to the emotional degree of mutual care and love among family members (35). When a patient feels the care provided by family, he or she will want to restore the family and social functions more, which will stimulate the patient to pay attention to self-integration in the disease, including diet control, exercise management, and adherence to medication. This suggests that in psychological nursing, we should pay attention to the cultivation of feelings between family members, and the strength of family affection is an important factor to help patients overcome the disease. F5 “Resolve” has the strongest positive correlation with M3 “Seeking social support.” The pain of the disease can affect important basic relationships and reduce the intimacy between family members, thus reducing patients' motivation to seek social support (54). Resolve refers to the degree of sharing of time, space, money and other aspects among family members (35). The essence of social support is an intimate relationship. Individuals can obtain social support such as emotional dependence, material help and spiritual companionship in the relationship with good resolution. This suggests that medical staff should help to strengthen the resolve between patients and their families through communicating better, hugging each other and participating in group activities together, strengthen individuals' active use of social support, and improve their self-management ability.

In the whole network, F3 “Growth” of family function and M3 “Seeking social support” of self-management have the greatest bridge expected influence. This shows that these two variables play the most important role in activating and maintaining the network composed of five components of family function and four components of self-management. In other words, as a bridge between family function and self-management, these two nodes may provide a new idea from the perspective of family function for interventions to improve self-management among patients with early CKD during the COVID-19 pandemic. Therefore, to improve patients' self-management, the most effective way is to improve growth and the motivation of patients to seek social support. During the pandemic, patients spent more time with their families, and a large part of the growth of patients comes from the support and guidance of other family members (55). Full support, correct guidance and continuous supervision can make patients aware of the importance of seeking social support and make them understand that they need to cooperate with their families to better self-management skills. Therefore, in clinical health education for patients with early CKD and their families during the COVID-19 pandemic, family members should be encouraged to accompany, understand and identify with patients, and enhance their sense of recognition and support. When introducing disease-related knowledge and how to carry out self-management, both patients and family members should be informed at the same time so that family members can fully understand the development and management of the disease and help family members can help supervise and guide patients in their daily lives (56). At the same time, patients should also be encouraged to actively express their feelings to their families, communicate more with them, and reduce the negative emotions that they are afraid will increase the burden on their families. With the joint efforts of patients and their families, patients can constantly improve their abilities to self-manage in a harmonious and positive family atmosphere to effectively alleviate the progress of disease and maintain good renal function.

There are some limitations in our study. First, the subjects of this study were all CKD patients from China, which may limit the generalization of our conclusions. Moreover, this network examines the influence between subjects at the group level, but whether the network results of individuals will be affected in the same way has not been determined. Future research needs to expand the sample size and carry out multicenter research. Second, a questionnaires survey was used in this study. The subjects' self-reported data may be affected by common method bias and subjective response bias, which may exaggerate the relationship between variables. Some objective clinical measurement data can be developed in future research. Third, this study is a cross-sectional study, and it is impossible to draw causal relationships from the current results. A cross-sectional study cannot determine the direction of edges in the network, which only indicates that the two variables are correlated but cannot determine causal effects. In future studies, longitudinal design can be carried out for research, or a direct acyclic graph (DAG) can be adopted to explore the potential causal relationship between family function and self-management, as long as the collected data are consistent with the hypothesis put forward by the DAG (57, 58). Fourth, the intervention targets identified in this study were based on the theory of network analysis, and we need to conduct case-control trials to test them in the next step.

## 5. Conclusion

Overall, this study used the method of network analysis for the first time to clarify the potential pathway between different components of family function and self-management among patients with early CKD during the COVID-19 pandemic and revealed a finer-grained relationship between them. More importantly, the bridge expected influence also provided a certain theoretical basis and scientific reference (i.e., promoting growth and initiative to seek social support) for intervention methods to improve patients' self-management abilities from the perspective of family function, enriched clinical psychological intervention, and contribute to delaying the progression of CKD.

## Data availability statement

The raw data supporting the conclusions of this article will be made available by the authors, without undue reservation.

## Ethics statement

The studies involving human participants were reviewed and approved by the Ethics Committee of the Second Affiliated Hospital of Air Force Medical University approved this study. The patients/participants provided their written informed consent to participate in this study.

## Author contributions

YC and YZ conceived and designed the study. RL, HW, SJ, and HL were responsible for collecting the data. TY analyzed the data. YC, TY, and NL wrote the paper. YZ, HL, and XL critically reviewed drafts of the paper and made constructive suggestions. All authors revised subsequent versions of the paper and approved the submitted version.

## Supplementary material

Supplementary Table 1 The correlation matrix of the network of family function and self-management.

Supplementary Figure 1 The accuracy of edge weights.

Supplementary Figure 2 The significance test of the difference in edge weight.

Supplementary Figure 3 The stability test of bridge expected influence.

Supplementary Figure 4 The significance test of the difference in BEI indices.

## References

[1] Evans M Lewis RD Morgan AR Whyte MB Hanif W Bain SC. A narrative review of chronic kidney disease in clinical practice: current challenges and future perspectives. Adv Ther. (2022) 39:3933–43. 10.1007/s12325-021-01927-z 34739697 PMC8569052

[2] Beng-Ongey H Robinson JS Moxey-Mims M. Chronic kidney disease emerging trends in children and what to do about it. J Natl Med Assoc. (2022) 114:S50–5. 10.1016/j.jnma.2022.05.002 35660045

[3] Hill NR Fatoba ST Oke JL Hirst JA O'Callaghan CA Lasserson DS. Global prevalence of chronic kidney disease–a systematic review and meta-analysis. PLoS ONE. (2016) 11:e0158765. 10.1371/journal.pone.0158765 27383068 PMC4934905

[4] Wang SZ Zhu YJ Tang WZ Liu N Ye F Li GQ. Prevalence of chronic kidney disease in Chinese adults and elderly population: a meta-analysis. Chinese J Gastroenterol. (2017) 37:5384–88. 10.3969/j.issn.1005-9202.2017.21.076

[5] Ozturk S Turgutalp K Arici M Odabas AR Altiparmak MR Aydin Z. Mortality analysis of COVID-19 infection in chronic kidney disease, haemodialysis and renal transplant patients compared with patients without kidney disease: a nationwide analysis from Turkey. Nephrol Dial Transplant. (2020) 35:2083–95. 10.1093/ndt/gfaa271 33275763 PMC7716804

[6] Peng S He J Huang J Lun L Zeng J Zeng S. Self-management interventions for chronic kidney disease: a systematic review and meta-analysis. BMC Nephrol. (2019) 20:142. 10.1186/s12882-019-1309-y 31027481 PMC6486699

[7] Jdiaa SS Mansour R El Alayli A Gautam A Thomas P Mustafa RA. COVID-19 and chronic kidney disease: an updated overview of reviews. J Nephrol. (2022) 35:69–85. 10.1007/s40620-021-01206-8 35013985 PMC8747880

[8] Nguyen NT Douglas C Bonner A. Effectiveness of self-management programme in people with chronic kidney disease: a pragmatic randomized controlled trial. J Adv Nurs. (2019) 75:652–64. 10.1111/jan.13924 30537153

[9] Donald M Kahlon BK Beanlands H Straus S Ronksley P Herrington G. Self-management interventions for adults with chronic kidney disease: a scoping review. BMJ Open. (2018) 8:e019814. 10.1136/bmjopen-2017-019814 29567848 PMC5875600

[10] Lin CC Anderson RM Chang CS Hagerty BM Loveland-Cherry CJ. Development and testing of the diabetes self-management Instrument: a confirmatory analysis. Res Nurs Health. (2008) 231:370–80. 10.1002/nur.20258 18213627

[11] Adejumo OA Okaka EI Akinbodewa AA Iyawe OI Edeki IR Abolarin OS. Self-perceived burden on caregivers, anxiety and depression among chronic kidney disease patients in Southern Nigeria. West Afr J Med. (2021) 38:335–41. 33900716

[12] Chuang LM Wu SV Lee MC Lin LJ Liang SY Lai PC. The effects of knowledge and self-management of patients with early-stage chronic kidney disease: self-efficacy is a mediator. Jpn J Nurs Sci. (2021) 18:e12388. 10.1111/jjns.12388 33174678

[13] Bennich BB Munch L Overgaard D Konradsen H Knop FK Røder M. Experience of family function, family involvement, and self-management in adult patients with type 2 diabetes: a thematic analysis. J Adv Nurs. (2020) 76:621–31. 10.1111/jan.14256 31670404

[14] Zhang X Zheng Y Qiu C Zhao Y Zang X. Well-being mediates the effects of social support and family function on self-management in elderly patients with hypertension. Psychol Health Med. (2020) 25:559–71. 10.1080/13548506.2019.1687919 31687846

[15] Cui Y Li R Yang T Wang H Jin S Liu N. Influence of positive and negative affect on self-management among patients with early chronic kidney disease during the COVID-19 pandemic: the mediating and suppressing effect of ego depletion. Front Psychiatry. (2022) 13:992404. 10.3389/fpsyt.2022.992404 36245863 PMC9556950

[16] Sacre JW Holmes-Truscott E Salim A Anstey KJ Drummond GR Huxley RR. Impact of the COVID-19 pandemic and lockdown restrictions on psychosocial and behavioural outcomes among Australian adults with type 2 diabetes: findings from the PREDICT cohort study. Diabet Med. (2021) 38:e14611. 10.1111/dme.14611 34053106 PMC8237067

[17] Lv H Meng J Chen Y Yang F Wang W Wei G. Impact of COVID-19 pandemic on elevated anxiety symptoms of maintenance hemodialysis patients in China: a one-year follow-up study. Front Psychiatry. (2022) 13:864727. 10.3389/fpsyt.2022.864727 35664473 PMC9160521

[18] Souza Júnior EV Viana ER Cruz DP Silva CDS Rosa RS Siqueira LR. Relationship between family functionality and the quality of life of the elderly. Rev Bras Enferm. (2021) 75:e20210106. 10.1590/0034-7167-2021-0106 34614103

[19] Barreto Andrade DM Montargil Rocha R Santos Ribeiro IJ. Depressive symptoms and family functionality in the elderly with diabetes mellitus. Issues Ment Health Nurs. (2020) 41:54–8. 10.1080/01612840.2019.1636167 31545908

[20] Lan X Lu X Yi B Chen X Jin S. Factors associated with self-management behaviors of patients with chronic obstructive pulmonary disease. Jpn J Nurs Sci. (2022) 19:e12450. 10.1111/jjns.12450 34398525

[21] Wensu Z Xidi Z Shaojie L Baohua Z Yunhan Y Huilan X. Does the presence of anxiety and depression symptoms mediate the association between family functions and self-efficacy in pregnant women in the third trimester? A community-based cross-sectional survey. Front Psychiatry. (2021) 12:726093. 10.3389/fpsyt.2021.726093 34803756 PMC8599816

[22] Santos DGMD Pallone JM Manzini CSS Zazzetta MS Orlandi FS. Relationship between frailty, social support and family functionality of hemodialysis patients: a cross-sectional study. Sao Paulo Med J. (2021) 139:570–5. 10.1590/1516-3180.2021.0089.r1.0904221 34706049 PMC9634838

[23] Galderisi S Rucci P Kirkpatrick B Mucci A Gibertoni D Rocca P. Italian network for research on psychoses. Interplay among psychopathologic variables, personal resources, context-related factors, and real-life functioning in individuals with schizophrenia: a network analysis. JAMA Psychiatry. (2018) 75:396–404. 10.1001/jamapsychiatry.2017.4607 29450447 PMC5875306

[24] Ren L Wang Y Wu L Wei Z Cui LB Wei X. Network structure of depression and anxiety symptoms in Chinese female nursing students. BMC Psychiatry. (2021) 21:279. 10.1186/s12888-021-03276-1 34059013 PMC8168020

[25] Fried EI van Borkulo CD Cramer AO Boschloo L Schoevers RA Borsboom D. Mental disorders as networks of problems: a review of recent insights. Soc Psychiatry Psychiatr Epidemiol. (2017) 52:1–10. 10.1007/s00127-016-1319-z 27921134 PMC5226976

[26] Fried EI Cramer AOJ. Moving forward: challenges and directions for psychopathological network theory and methodology. Perspect Psychol Sci. (2017) 12:999–1020. 10.1177/1745691617705892 28873325

[27] Contreras A Nieto I Valiente C Espinosa R Vazquez C. The study of psychopathology from the network analysis perspective: a systematic review. Psychother Psychosom. (2019) 88:71–83. 10.1159/000497425 30889609

[28] Haslbeck JMB Fried EI. How predictable are symptoms in psychopathological networks? A reanalysis of 18 published datasets. Psychol Med. (2017) 47:2767–76. 10.1017/S0033291717001258 28625186

[29] Luo D Xu JJ Cai X Zhu M Wang H Yan D. The effects of family functioning and resilience on self-management and glycaemic control among youth with type 1 diabetes. J Clin Nurs. (2019) 28:4478–87. 10.1111/jocn.15033 31410916

[30] Wu L Ren L Wang Y Zhang K Fang P Liu X. The item network and domain network of burnout in Chinese nurses. BMC Nurs. (2021) 20:147. 10.1186/s12912-021-00670-8 34404401 PMC8369754

[31] Epskamp S Fried EI. A tutorial on regularized partial correlation networks. Psychol Methods. (2018) 23:617–34. 10.1037/met0000167 29595293

[32] Jones PJ Ma R McNally RJ. Bridge centrality: a network approach to understanding comorbidity. Multivariate Behav Res. (2021) 56:353–67. 10.1080/00273171.2019.1614898 31179765

[33] Kendall M. Multivaraiate Anlysis. London: Charles Griffin and Company Limited (1975).

[34] Smilkstein G. The family APGAR: a proposal for a family function test and its use by physicians. J Fam Pract. (1978) 6:1231–9. 660126

[35] Lv F Gu Y. Family APGAR questionnaire and its clinical application. Hospital Management Forum. (1995) 02:56–9.20849630

[36] Lin CC Wu CC Wu LM Chen HM Chang SC. Psychometric evaluation of a new instrument to measure disease self-management of the early stage chronic kidney disease patients. J Clin Nurs. (2013) 22:1073–9. 10.1111/j.1365-2702.2011.04048.x 22642723

[37] Liu YJ Jia Q Xu HL Ji M Ma LL. Revision of the Taiwan instrument of early stage chronic kidney disease self-management. J Nurs Sci. (2015) 30:18–21.

[38] Epskamp S Cramer AOJ Waldorp LJ Schmittmann VD Borsboom D. Qgraph: network visualizations of relationships in psychometric data. J Stat Softw. (2012) 48:1–18. 10.18637/jss.v048.i04

[39] Epskamp S Borsboom D Fried EI. Estimating psychological networks and their accuracy: a tutorial paper. Behav Res Methods. (2018) 50:195–212. 10.3758/s13428-017-0862-1 28342071 PMC5809547

[40] Tibshirani R. Regression shrinkage and selection via the lasso: a retrospective. J R Stat Soc B. (2011) 73:273–82. 10.1111/j.1467-9868.2011.00771.x34741355

[41] Chen JH Chen ZH. Extended Bayesian information criteria for model selection with large model spaces. Biometrika. (2008) 95:759–71. 10.1093/biomet/asn034

[42] Psihogios AM Daniel LC Tarazi R Smith-Whitley K Patterson CA Barakat LP. Family functioning, medical self-management, and health outcomes among school-aged children with sickle cell disease: a mediation model. J Pediatr Psychol. (2018) 43:423–33. 10.1093/jpepsy/jsx120 29048590 PMC6927851

[43] Thompson G McBride RB Hosford CC Halaas G. Resilience among medical students: the role of coping style and social support. Teach Learn Med. (2016) 28:174–82. 10.1080/10401334.2016.1146611 27064719

[44] Al-Dwaikat TN Rababah JA Al-Hammouri MM Chlebowy DO. Social support, self-efficacy, and psychological wellbeing of adults with type 2 diabetes. West J Nurs Res. (2021) 43:288–97. 10.1177/0193945920921101 32419665

[45] Kiesswetter M Marsoner H Luehwink A Fistarol M Mahlknecht A Duschek S. Impairments in life satisfaction in infertility: associations with perceived stress, affectivity, partnership quality, social support and the desire to have a child. Behav Med. (2020) 46:130–41. 10.1080/08964289.2018.1564897 30726170

[46] Meuleman Y Ten Brinke L Kwakernaak AJ Vogt L Rotmans JI Bos WJ. Perceived barriers and support strategies for reducing sodium intake in patients with chronic kidney disease: a qualitativestudy. Int J Behav Med. (2015) 22:530–9. 10.1007/s12529-014-9447-x 25298022

[47] Balcells-Balcells A Giné C Guàrdia-Olmos J Summers JA Mas JM. Impact of supports and partnership on family quality of life. Res Dev Disabil. (2019) 85:50–60. 10.1016/j.ridd.2018.10.006 30468989

[48] Halder MM Wakefield JR Bowe M Kellezi B Mair E McNamara N. Evaluation and exploration of a social prescribing initiative: study protocol. J Health Psychol. (2021) 26:345–56. 10.1177/1359105318814160 30488733

[49] Haslam D Mejia A Thomson D Betancourt T. Self-regulation in low- and middle-income countries: challenges and future directions. Clin Child Fam Psychol Rev. (2019) 22:104–17. 10.1007/s10567-019-00278-0 30725308

[50] Vaid E Lansing AH Stanger C. Problems with self-regulation, family conflict, and glycemic control in adolescents experiencing challenges with managing type 1 diabetes. J Pediatr Psychol. (2018) 43:525–33. 10.1093/jpepsy/jsx134 29077875 PMC5961369

[51] Shah SMA Mohammad D Qureshi MFH Abbas MZ Aleem S. Prevalence, psychological responses and associated correlates of depression, anxiety and stress in a global population, during the coronavirus disease (COVID-19) pandemic. Community Ment Health J. (2021) 57:101–10. 10.1007/s10597-020-00728-y 33108569 PMC7590908

[52] Chen Y Li M Zhou L Chen C Li N Meng J. Association among sleep, depression, and health related quality of life in patients with non-dialysis chronic kidney disease during the coronavirus disease 2019 pandemic. Ann Palliat Med. (2022) 11:1865–75. 10.21037/apm-21-3416 35272471

[53] Leukel PJ Kollin SR Lewis BR Lee AA. The influence of emotion regulation and family involvement on diabetes distress among adults with type 2 diabetes. J Behav Med. (2022) 10:1–10. 10.1007/s10865-022-00351-0 35948697 PMC9364847

[54] Smith AA. Intimacy and family relationships of women with chronic pain. Pain Manag Nurs. (2003) 4:134–42. 10.1016/S1524-9042(03)00030-4 14566711

[55] Whitehead L Jacob E Towell A Abu-Qamar M Cole-Heath A. The role of the family in supporting the self-management of chronic conditions: a qualitative systematic review. J Clin Nurs. (2018) 27:22–30. 10.1111/jocn.13775 28231630

[56] Daniel O Dest A Munson A Pulley DV Sadeghzadeh C Golin C. Interventions to enhance patient and family engagement among adults with multiple chronic conditions: a systematic scoping review. Med Care. (2020) 58:407–16. 10.1097/MLR.0000000000001274 31914106

[57] Moffa G Catone G Kuipers J Kuipers E Freeman D Marwaha S. Using directed acyclic graphs in epidemiological research in psychosis: an analysis of the role of bullying in psychosis. Schizophr Bull. (2017) 43:1273–9. 10.1093/schbul/sbx013 28521005 PMC5737513

[58] Rohrer JM. Thinking clearly about correlations and causation: graphical causal models for observational data. Adv Meth Pract Psych. (2018) 1:27–42. 10.1177/2515245917745629

