# Supplementary material for: Network structure of family function and self-management in patients with early chronic kidney disease amid the COVID-19 pandemic
Source: Front Public Health. 2023 Jan 10;10:1073409. doi: 10.3389/fpubh.2022.1073409 (PMC9871502; doi:10.3389/fpubh.2022.1073409)
Supplement: Supplementary Table 1 — The correlation matrix of the network of family function and self-management. [file Table_1.DOCX]

Table S1 The correlation matrix of the network of family function and self-management

|  | M1 | M2 | M3 | M4 | F1 | F2 | F3 | F4 | F5 |
| --- | --- | --- | --- | --- | --- | --- | --- | --- | --- |
| M1 |  | 0.596 | 0.109 | 0.142 | 0.000 | 0.000 | 0.090 | 0.045 | 0.000 |
| M2 | 0.596 |  | 0.463 | 0.002 | 0.000 | -0.028 | 0.010 | 0.000 | 0.015 |
| M3 | 0.109 | 0.463 |  | 0.000 | 0.012 | 0.074 | 0.000 | 0.019 | 0.056 |
| M4 | 0.142 | 0.002 | 0.000 |  | 0.000 | 0.000 | 0.016 | 0.031 | 0.012 |
| F1 | 0.000 | 0.000 | 0.012 | 0.000 |  | 0.465 | 0.138 | 0.175 | 0.000 |
| F2 | 0.000 | -0.028 | 0.074 | 0.000 | 0.465 |  | 0.116 | 0.136 | 0.252 |
| F3 | 0.090 | 0.010 | 0.000 | 0.016 | 0.138 | 0.116 |  | 0.253 | 0.239 |
| F4 | 0.045 | 0.000 | 0.019 | 0.031 | 0.175 | 0.136 | 0.253 |  | 0.396 |
| F5 | 0.000 | 0.015 | 0.056 | 0.012 | 0.000 | 0.252 | 0.239 | 0.396 |  |
