# Supplementary figures and images for: Network structure of family function and self-management in patients with early chronic kidney disease amid the COVID-19 pandemic
Source: Front Public Health. 2023 Jan 10;10:1073409. doi: 10.3389/fpubh.2022.1073409 (PMC9871502; doi:10.3389/fpubh.2022.1073409)

● Bootstrap mean    ● Sample

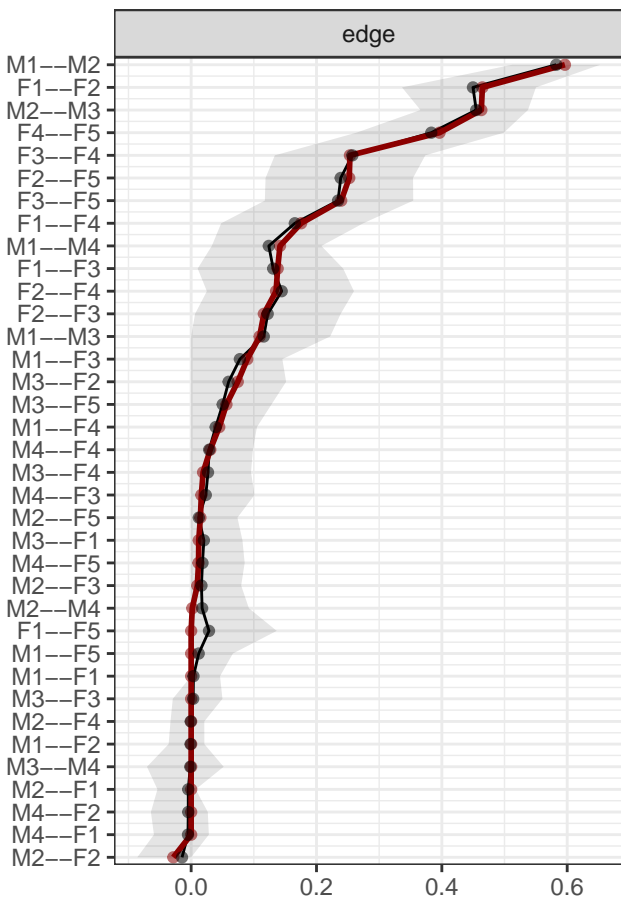

Supplement: Supplementary Figure 1 — The accuracy of edge weights. [file Image_1.PDF]

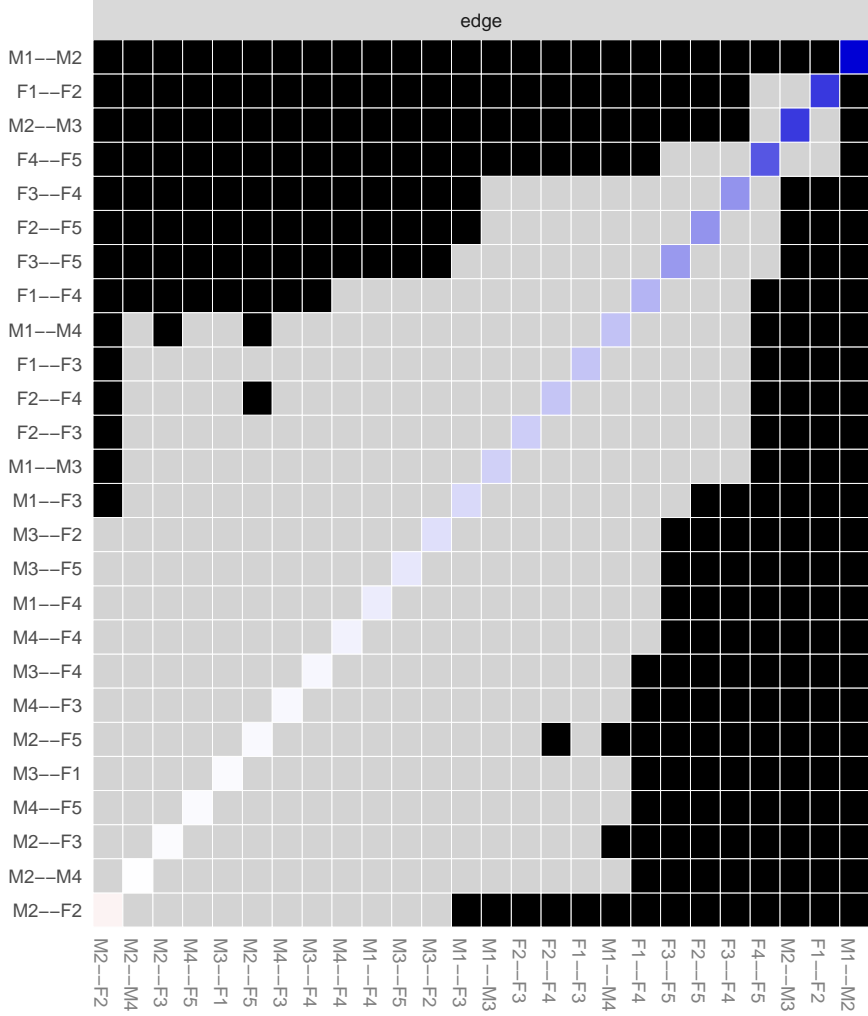

Supplement: Supplementary Figure 2 — The significance test of the difference in edge weight. [file Image_2.PDF]

bridgeExpectedInfluence

Average correlation with original sample

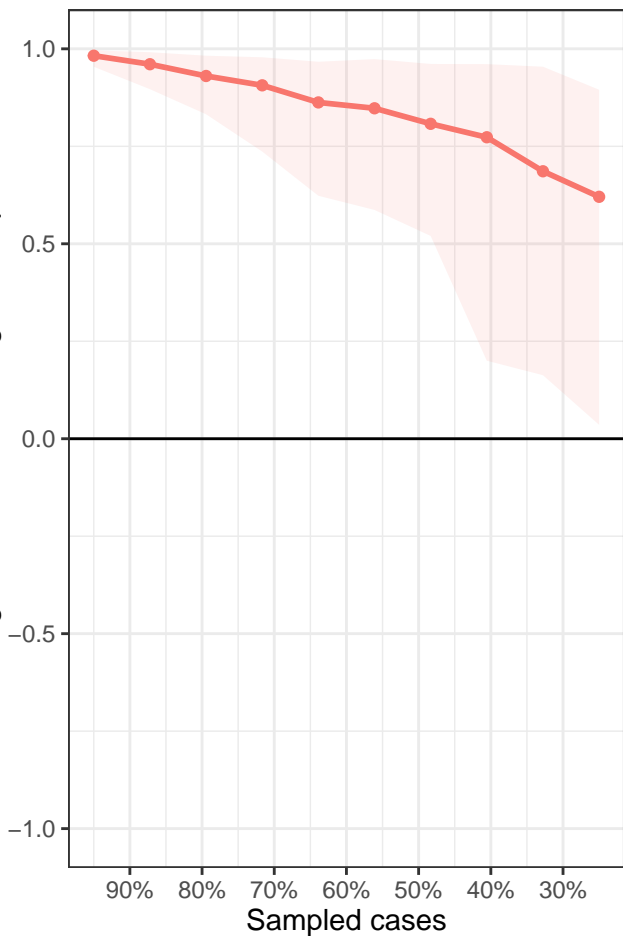

Supplement: Supplementary Figure 3 — The stability test of bridge expected influence. [file Image_3.PDF]

bridgeExpectedInfluence

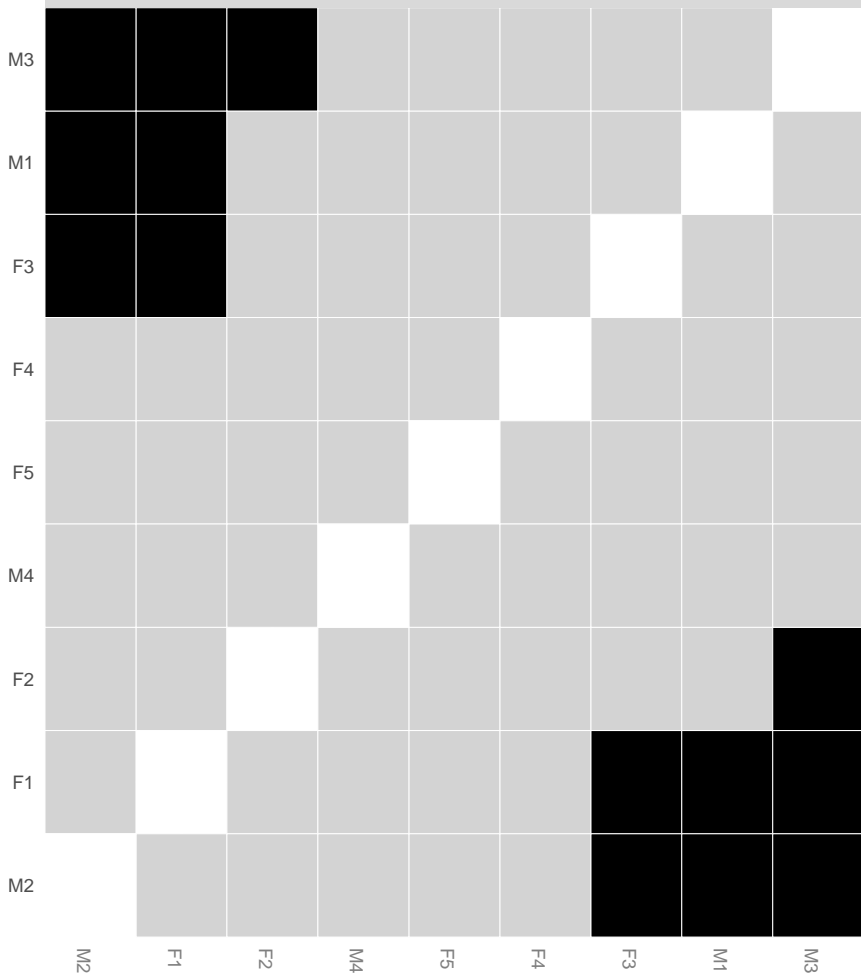

Supplement: Supplementary Figure 4 — The significance test of the difference in BEI indices. [file Image_4.PDF]
